# Geometric and energetic data from *ab initio* calculations of haloethene, haloimine, halomethylenephosphine, haloiminophosphine, halodiazene, halodiphosphene and halocyclopropane

**DOI:** 10.1016/j.dib.2019.104738

**Published:** 2019-11-02

**Authors:** Kridtin Chinsukserm, Wanutcha Lorpaiboon, Peerayar Teeraniramitr, Taweetham Limpanuparb

**Affiliations:** a Mahidol University International College, Salaya, Nakhon Pathom 73170, Thailand; b Mahidol University International Demonstration School, Salaya, Nakhon Pathom 73170, Thailand

**Keywords:** *cis* effect, Haloethene, Haloimine, Halomethylenephosphine, Haloiminophosphine, Halodiazene, Halodiphosphene, Halocyclopropane

## Abstract

This article presents theoretical data on geometric and energetic features of halogenated compounds of ethene (C=C), imine (C=N), methylenephosphine (C=P), iminophosphine (N=P), diazene (N=N), diphosphene (P=P) and cyclopropane (Δ). The data were obtained from *ab initio* geometric optimization and frequency calculations at HF, B3LYP, MP2 and CCSD levels of theory on 6–311++G(d,p) basis set. Input structures were generated by shell scripts and run by Q-Chem quantum chemical package. The output files were processed to extract geometric and energetic information by Wolfram Mathematica.

**Table:** Specifications Table

Subject area	Chemistry
More specific subject area	Physical and Theoretical Chemistry/Spectroscopy
Type of data	Tables/Q-Chem output files
How data was acquired	Quantum chemical computation
Data format	Both raw and analyzed
Parameters for data collection	HF/6-311++G(d,p), B3LYP/6-311++G(d,p), MP2/6-311++G(d,p) and CCSD/6-311++G(d,p)
Description of data collection	Q-Chem 5.1, Developer Version
Data source location	Thailand
Data accessibility	With the article

**Table:** 

**Value of the Data** • Systematic and high-quality quantum chemical results in this article can be used by scientists to understand the nature of chemical bonding in these compounds and to explore special phenomena such as the *cis* effect [1]. • General/trivial trends and some anomalies can be observed in both geometric and energetic data of the compounds. Experimental results are currently very limited and these trends can provide further insights into development of future experiments. • Provided source codes can be modified for uses in other classes of compounds.

## 1 Data

In this data set, we present the theoretical results from a combinatorial investigation of substituted cyclopropane and double-bonded (a combination of C, N and P) compounds. The data in this paper were generated and optimized in vacuum by *ab initio* quantum chemical calculations at HF/6-311++G(d,p), B3LYP/6-311++G(d,p), MP2/6-311++G(d,p) and CCSD/6-311++G(d,p) levels of calculations.

• The geometric data include all the available bond lengths of A_1_=A_2_ and A_1_-α, all bond angles of α-A_1_-β and α-A_1_-A_2_, and dihedral angles of α-A_1_-A_2_-β, where A_1_/A_2_ and α/β refer to the central and peripheral atoms respectively.
• The energetic data include electronic energy (*E*_elec_), thermal correction to enthalpy (*H*_corr_), enthalpy (*H*), entropy (*S*), and Gibbs free energy at 298.15 K (*G*).
• The data are available in tables (.xlsx files) along with other associated Unix shell scripts (as text files) and Wolfram Mathematica notebooks (.nb files) are provided in the supplementary information.
• Output files which include vibrational spectrum are also available and can be viewed in IQmol [2].

Here we include geometric and energetic data of the halogenated forms of seven classes of compounds: ethene (C=C), imine (C=N), methylenephosphine (C=P), iminophosphine (N=P), diazene (N=N), diphosphene (P=P) and cyclopropane (Δ) where substitutions are via halogenation (including F, Cl, Br and I) with all degrees of substitution from mono- to tetra-substitution. The total numbers of all possible compounds are as follows: 175 for C=C (Table 1), 125 for C=N (Table 2), 125 for C=P (Table 3), 50 for N=P (Table 4), 30 for N=N (Table 5), 30 for P=P (Table 6) and 315 for Δ (Table 7). The total number of structures are summarized in Table 8. The dataset described in this paper is the most comprehensive compared to other previously published results on these compounds [[3], [4], [5]].

**Table 1:** List of 175 structures for haloethene (C=C).

Empirical Formula	Number of Empirical Formulae	Structure	Total number of structures
C_2_α_4_	$\left(\begin{array}{c} 5\\ 1 \end{array}\right)=5$	α_2_C=Cα_2_	5 × 1 = 5
C_2_α_3_β	$\left(\begin{array}{c} 5\\ 1 \end{array}\right)\left(\begin{array}{c} 4\\ 1 \end{array}\right)=20$	α_2_C=Cαβ	20 × 1 = 20
C_2_α_2_β_2_	$\left(\begin{array}{c} 5\\ 2 \end{array}\right)=10$	αβC=Cβα (*E*/*Z*)	10 × 3 = 30
		α_2_C=Cβ_2_	
C_2_α_2_βγ	$\left(\begin{array}{c} 5\\ 1 \end{array}\right)\left(\begin{array}{c} 4\\ 2 \end{array}\right)=30$	αβC=Cαγ (*E*/*Z*)	30 × 3 = 90
		α_2_C=Cβγ	
C_2_αβγδ	$\left(\begin{array}{c} 5\\ 4 \end{array}\right)=5$	αβC=Cγδ (*E*/*Z*)	5 × 6 = 30
		αγC=Cβδ (*E*/*Z*)	
		αδC=Cβγ (*E*/*Z*)

**Table 2:** List of 125 structures for haloimine (C=N).

Empirical Formula	Number of Empirical Formulae	Structure	Total number of structures
CNα_3_	$\left(\begin{array}{c} 5\\ 1 \end{array}\right)\;=5\;$	α_2_C=Nα	5 × 1 = 5
CNα_2_β	$\left(\begin{array}{c} 5\\ 1 \end{array}\right)\left(\begin{array}{c} 4\\ 1 \end{array}\right)=20\;$	α_2_C = Nβ αβC = Nα (*E*/*Z*)	20 × 3 = 60
CNαβγ	$\left(\begin{array}{c} 5\\ 3 \end{array}\right)=10\;$	αβC=Nγ (*E*/*Z*) αγC=Nβ (*E*/*Z*) βγC=Nα (*E*/*Z*)	10 × 6 = 60

**Table 3:** List of 125 structures for halomethylenephosphine (C=P).

Empirical Formula	Number of Empirical Formulae	Structure	Total number of structures
CPα_3_	$\left(\begin{array}{c} 5\\ 1 \end{array}\right)\;=5\;$	α_2_C=Pα	5 × 1 = 5
CPα_2_β	$\left(\begin{array}{c} 5\\ 1 \end{array}\right)\left(\begin{array}{c} 4\\ 1 \end{array}\right)=20\;$	α_2_C=Pβ αβC = Pα (*E*/*Z*)	20 × 3 = 60
CPαβγ	$\left(\begin{array}{c} 5\\ 3 \end{array}\right)=10\;$	αβC=Pγ (*E*/*Z*) αγC=Pβ (*E*/*Z*) βγC=Pα (*E*/*Z*)	10 × 6 = 60

**Table 4:** List of 50 structures for haloiminophosphine (N=P).

Empirical Formula	Number of Empirical Formulae	Structure	Total number of structures
NPα_2_	$\left(\begin{array}{c} 5\\ 1 \end{array}\right)\;=5\;$	αN=Pα (*E*/*Z*)	5 × 2 = 10
NPαβ	$\left(\begin{array}{c} 5\\ 2 \end{array}\right)=10$	αN=Pβ (*E*/*Z*) βN=Pα (*E*/*Z*)	10 × 4 = 40

**Table 5:** List of 30 structures for halodiazene (N=N).

Empirical Formula	Number of Empirical Formulae	Structure	Total number of structures
N_2_α_2_	$\left(\begin{array}{c} 5\\ 1 \end{array}\right)\;=5$	αN=Nα (*E/Z*)	5 × 2 = 10
N_2_αβ	$\left(\begin{array}{c} 5\\ 2 \end{array}\right)\;=\;10$	αN=Nβ (*E/Z*)	10 × 2 = 20

**Table 6:** List of 30 structures for halodiphosphene (P=P).

Empirical Formula	Number of Empirical Formulae	Structure	Total number of structures
P_2_α_2_	$\left(\begin{array}{c} 5\\ 1 \end{array}\right)\;=5$	αP=Pα (*E*/*Z*)	5 × 2 = 10
P_2_αβ	$\left(\begin{array}{c} 5\\ 2 \end{array}\right)\;=\;10$	αP=Pβ (*E*/*Z*)	10 × 2 = 20

**Table 7:** List of 315 structures for halocyclopropane (Δ).

Empirical Formula	Number of Empirical Formulae	Structure	Total number of structures
(CH_2_)C_2_α_4_	$\left(\begin{array}{c} 5\\ 1 \end{array}\right)=5$	α_2_Δα_2_	5 × 1 = 5
(CH_2_)C_2_α_3_β	$\left(\begin{array}{c} 5\\ 1 \end{array}\right)\left(\begin{array}{c} 4\\ 1 \end{array}\right)=20$	α_2_Δαβ (*R*/*S*)^a^	16 × 2 = 32 (α is not H.)
			4 × 1 = 4 (α is H.)
(CH_2_)C_2_α_2_β_2_	$\left(\begin{array}{c} 5\\ 2 \end{array}\right)=10$	α_2_Δβ_2_	10 × 4 = 40
		αβΔαβ (*E*, *R*/*S*)	
		αβΔαβ (*Z*, meso isomer)	
(CH_2_)C_2_α_2_βγ	$\left(\begin{array}{c} 5\\ 1 \end{array}\right)\left(\begin{array}{c} 4\\ 2 \end{array}\right)=30$	αβΔαγ (*E*/*Z*, *R*/*S*)	24 × 6 = 144 (α is not H.)
		α_2_Δβγ (*R*/*S*)^a^	6 × 5 = 30 (α is H.)
(CH_2_)C_2_αβγδ	$\left(\begin{array}{c} 5\\ 4 \end{array}\right)=5$	αβΔγδ (*E*/*Z*, *R*/*S*)	5 × 12 = 60
		αγΔβδ (*E*/*Z, R*/*S*)	
		αδΔβγ (*E*/*Z*, *R*/*S*)	

a If α is H there is no *R*/*S* and the number of total isomers must be calculated separately for this case.

**Table 8:** A summary of all data in this paper.^a^

System	Number of isomers				HF and B3LYP		MP2		CCSD	
	*E*	*Z*	other	total	opt	freq	opt	freq	opt	freq
C=C (ethene)	55	55	65	175	all	all	all	all	all	30
C=N (imine)	50	50	25	125	all	all	all	all	all	all
C=P (methylenephosphine)	50	50	25	125	all	all	all	all	all	all
N=P (iminophosphine)	25	25	0	50	all	all	all	all	all	all
N=N (diazene)	15	15	0	30	all	all	all^b^	all^b^	all^b^	all^b^
P=P (diphosphene)	15	15	0	30	all	all	all	all	all	all
Δ (cyclopropane)	110	100	105	315	all^c^	all^c^	all^c^	all^c^	all^c^	20^c^

a Opt and freq stand for geometry optimization and frequency calculation respectively.

b Some structures are unbound.(26 converged structures for MP2 and CCSD).

c Excluding enantiomers, there are 5 + 20 + 10 × 3 + 30 × 3 + 5 × 6 = 175 halocyclopropane structures which is the same as the number of structures for ethene family.

## 2 Experimental design, materials, and methods

Q-Chem input files (.inp) were generated partly by using a Unix shell script previously described elsewhere [[6], [7], [8]]. The *ab initio* calculations were performed using the Q-Chem 5.1 program package [9] to optimize the structures at HF, B3LYP, MP2 and CCSD levels of theory on 6-311++G(d,p) basis set. The rationale behind the choosing of this basis set is the availability of iodine, which was used as a part to fulfill all possible halogenation in this combinatorial investigation. Frequency calculations confirm that structures are minima on the electronic potential energy surface for all HF, B3LYP and MP2 jobs and some CCSD jobs where possible. The output files were processed using Wolfram Mathematica [10] to extract relevant geometric and energetic data of all the seven classes of compounds in batches.
