# Supplementary material for: Geometric and energetic data from ab initio calculations of haloethene, haloimine, halomethylenephosphine, haloiminophosphine, halodiazene, halodiphosphene and halocyclopropane
Source: Data Brief. 2019 Nov 2;27:104738. doi: 10.1016/j.dib.2019.104738 (PMC6861602; doi:10.1016/j.dib.2019.104738)

1. Structure & file name convention

structure	file name
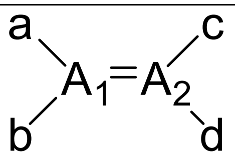	abA_1A_2cd<br>(in folder CC, CN, CP, NN, NP, PP)
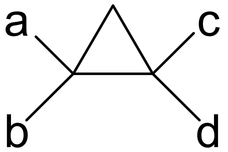	abCCCcd<br>(in folder CCC)

- A_1, A_2 are C, N, P.
- a, b, c, d are H, F, Cl, Br, I, E (a lone-pair of electrons).

2. Additional naming convention to uniquely identify isomers of an empirical formula

- CIP priority rules are used to determine *E/Z/G* (geminal) and to assign numbers starting at 1 to each of the substituents a, b, c, d.
- If two or more substituents are the same, they are given the same priority. The priority number of the next substituent(s) are incremented accordingly. For example, Cl F F H are assigned priority numbers 1 2 2 4 respectively.
- Lone pairs of electrons are assigned the lowest priority.
- The name of the compound is composed of *E/Z/G* followed by max(|pr(a)-pr(b)|, |pr(c)-pr(d)|) where pr is CIP priority number. The possible values are 0, 1, 2, 3 for *G* and 1, 2, 3 for *E/Z*.
- In the case of N=P where ambiguity exists, *E/Z* is followed by a if the lower priority substituent is bonded to the N (lower priority side) and b otherwise instead of number 2.

Example for C=C (empirical formulae, C_2BrFI_2, C_2BrClF_2, $C_2Cl_2F_2$, C_2F_3I, CI_4)

Z3 FICCClBr
E3 FICCBrCl
Z2 FBrCCClI
E2 BrFCCClI
Z1 ClFCCIBr
E1 ClFCCBrI

Z2 FClCCFBr
E2 BrFCCFCl
G1 FFCCBrCl

Z3 FICCClI
E3 FICCICl
G1 ClFCCII

Z2 FICCFI
E2 FICCIF
G0 FFCCII

G1 FFCCFI

G0 IICCII

Example for N=P (empirical formulae, FINP, NPX_2)

Za FENPIE
Ea FENPEI
Zb IENPFE
Eb IENPEF

Z2 XENPXE
E2 XENPEX

3. Basic shell scripts (works for GNU grep only)

Basic output processing

```
ls *out > 0_file_list.txt

grep "Final energy" *out | sed 's/\:\ Final\ energy \is/,/g' > 1_final_energy.csv

grep -m1 "Zero point vib" *out | sed 's/\:\ \ \ \Zero\ point\ vibrational\ energy\:/,/g' | sed 's/kcal\/mol//g'
    > 3_zero_point.csv

grep -m1 "Total Enthalpy" *out | sed 's/\:\ \ \ \Total\ Enthalpy\:/,/g' | sed 's/kcal\/mol//g' > 4_enthalpy.csv

grep -m1 "Total Entropy" *out | sed 's/\:\ \ \ \Total\ Entropy\:/,/g' | sed 's/cal\/mol\.K//g' > 5_entropy.csv
```

Generating geometry files

```
for i in *out; do fname=XYZ/${i%out}xyz; echo $i > $fname ; grep -A500 "CONVERGED" $i | grep -A500 -m1
    "Coordinates" | grep -B500 -m1 "end" >> $fname; done
```

Generating input files from existing converged structures

```
for i in *out; do fname=../MP2/${i%out}inp; echo $fname > $fname ; grep -A500 "CONVERGED" $i | grep -A500 -m1
    "molecule" | grep -B500 -m1 "end" >> $fname ; cat ../MP2template.txt >> $fname ; done
```

Generating a batch file to run on a server

```
for i in *inp; do fni=${i%inp}inp; fno=${i%inp}out; echo qchem -nt 6 $fni $fno >> batch.sh  ; done
```

4. Summary of CCSD electronic results

When are *Z* isomers more stable than *E* isomers?

C=C

C_2HBrFI	*Z1*
$C_2H_2F_2$	*Z2*
C_2H_2FI	*Z2*
$C_2H_2Cl_2$	*Z2*
C_2H_2ClF	*Z2*
C_2H_2BrF	*Z2*
C_2HF_2I	*Z2*
C_2HClF_2	*Z2*
C_2HBrF_2	*Z2*

C=N

CNBrFI	*Z1*
CNHFI	*Z1,Z2,Z3*
CNHClI	*Z1,Z2,Z3*
CNHClF	*Z1,Z2,Z3*
CNHBrI	*Z3*
CNHBrF	*Z1,Z2,Z3*
CNHBrCl	*Z1,Z2,Z3*
CNF_2I	*Z2*
$CNClF_2$	*Z2*
$CNBrF_2$	*Z2*
CNH_2I	*Z2*
CNH_2F	*Z2*
CNH_2Cl	*Z2*
CNH_2Br	*Z2*
$CNHF_2$	*Z3*
$CNHCl_2$	*Z3*
$CNHBr_2$	*Z3*

C=P

CPHFI	*Z1,Z2,Z3*
CPHClI	*Z2,Z3*
CPHClF	*Z2,Z3*
CPHBrF	*Z1,Z2,Z3*
CPHBrCl	*Z2,Z3*
$CPClF_2$	*Z2*
CPH_2I	*Z2*
CPH_2Cl	*Z2*
CPH_2Br	*Z2*
$CPHF_2$	*Z3*
$CPHCl_2$	*Z3*

N=N

N_2F_2	*Z2*
N_2FI	*Z2*
N_2Cl_2	*Z2*
N_2ClI	*Z2*
N_2ClF	*Z2*
N_2Br_2	*Z2*
N_2BrF	*Z2*
N_2BrCl	*Z2*
N_2HI	*Z2*
N_2HF	*Z2*
N_2HCl	*Z2*
N_2HBr	*Z2*

All

Except N_2I_2 which

is unbounded.

N=P

NPFI	*Za,Zb*
NPClI	*Za,Zb*
NPClF	*Za,Zb*
NPBrI	*Za,Zb*
NPBrF	*Za,Zb*
NPBrCl	*Za,Zb*
NPHI	*Za,Zb*
NPHF	*Zb*
NPHCl	*Zb*
NPHBr	*Zb*
NPI_2	*Z2*
NPF_2	*Z2*
$NPCl_2$	*Z2*
$NPBr_2$	*Z2*

P=P

None

Δ

C_3H_2HClFI	*Z1,Z2*
C_3H_2HBrFI	*Z1*
$C_3H_2HBrClI$	*Z1*
$C_3H_2HBrClF$	*Z1*
$C_3H_2HF_2I$	*Z2*
$C_3H_2HCl_2I$	*Z2*
$C_3H_2HCl_2F$	*Z3*
$C_3H_2HClF_2$	*Z2*
$C_3H_2HBr_2I$	*Z2*
$C_3H_2HBrF_2$	*Z2*

5. Sample output information

Compound	a	b	c	d	Bond length (Å)					Bond angle (°)						Dihedral angle (°)					Energy (Hartree)				
					aC	bC	cC	dC	CC	aCb	cCd	aCC	bCC	cCC	dCC	aCCc	aCCd	bCCc	bCCd		E_elec	H_corr	H	S	G
BrFCCClI	Br	F	Cl	I	1.869	1.306	1.721	2.101	1.315	111.90	116.68	126.68	121.41	122.34	120.98	180	0	0	180		-10123.8	0.024331	-10123.8	0.00014	-10123.9

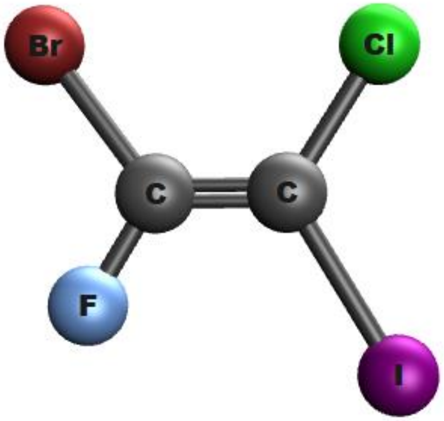

Supplement: Multimedia component 1 [file mmc1.pdf]
